# The association between telomere length and mortality in Bangladesh

**DOI:** 10.18632/aging.101246

**Published:** 2017-06-15

**Authors:** Samantha G. Dean, Chenan Zhang, Jianjun Gao, Shantanu Roy, Justin Shinkle, Mekala Sabarinathan, Maria Argos, Lin Tong, Alauddin Ahmed, Md. Tariqul Islam, Tariqul Islam, Muhammad Rakibuz-Zaman, Golam Sarwar, Hasan Shahriar, Mahfuzar Rahman, Md. Yunus, Joseph H. Graziano, Lin S. Chen, Farzana Jasmine, Muhammad G. Kibriya, Habibul Ahsan, Brandon L. Pierce

**Affiliations:** ^1^ Department of Public Health Sciences, University of Chicago, Chicago, IL 60637, USA; ^2^ Department of Epidemiology and Biostatistics, University of California, San Francisco, San Francisco, CA 94158, USA; ^3^ Department of Psychiatry, University of California San Diego, La Jolla, CA 92093, USA; ^4^ Division of Epidemiology and Biostatistics, University of Illinois at Chicago, Chicago, IL 60637, USA; ^5^ UChicago Research Bangladesh, Dhaka, Bangladesh; ^6^ Research and Evaluation Division, BRAC, Dhaka, Bangladesh; ^7^ International Centre for Diarrhoeal Disease Research, Dhaka, Bangladesh; ^8^ Department of Environmental Health Sciences, Mailman School of Public Health, Columbia University, New York, NY 10032, USA; ^9^ Department of Human Genetics, University of Chicago, Chicago, IL 60615, USA; ^10^ Comprehensive Cancer Center, University of Chicago, Chicago, IL 60615, USA; ^11^ Department of Medicine, University of Chicago, Chicago, IL 60615, USA; ^12^ Current address: Division of Foodborne, Waterborne, and Environmental Diseases, Center for Disease Control, Atlanta GA, 30333, USA

**Keywords:** telomere length, Bangladesh, mortality, arsenic, circulatory disease

## Abstract

Telomeres are tandem repeat sequences at the end of chromosomes that bind proteins to protect chromosome ends. Telomeres shorten with age, and shorter leukocyte telomere length (TL) has been associated with overall mortality in numerous studies. However, this association has not been tested in populations outside of Europe and the U.S. We assessed the association between TL and subsequent mortality using data on 744 mortality cases and 761 age-/sex-matched controls sampled from >27,000 participants from three longitudinal Bangladeshi cohorts: Health Effects of Arsenic Longitudinal Study (HEALS), HEALS Expansion (HEALS-E), and Bangladesh Vitamin E and Selenium Trial (BEST). We used conditional logistic regression to estimate odds ratios (ORs) for the association between a standardized TL variable and overall mortality, as well as mortality from chronic diseases, respiratory diseases, circulatory diseases, and cancer. In HEALS and BEST, we observed an association between shorter TL and increased overall mortality (P=0.03 and P=0.03), mortality from chronic disease (P=0.01 and P=0.03) and mortality from circulatory disease (P=0.03 and P=0.04). Results from pooled analyses of all cohorts were consistent with HEALS and BEST. This is the first study demonstrating an association between short TL and increased mortality in a population of non-European ancestry.

## INTRODUCTION

Telomeres are repeating TTAGGG nucleotide sequences at the end of human chromosomes. The telomere sequence binds a protein complex to form a cap that protects chromosome ends from damage. When cells divide and DNA replicates, some nucleotide content is lost from each telomere. As telomeres shorten, they eventually reach a critical length that triggers cell senescence or apoptosis. Because telomere length (TL) shortens with age in most human tissues, it is considered a potential biomarker of aging and a potential contributor to age-related diseases [1]. Prior studies indicate that shorter TL in leukocytes is associated with increasing age, male sex, and Caucasian race/ethnicity (as compared to African American) [1–3].

Shorter TL has been associated with increased overall mortality [2–5]. However, the literature on this topic is not entirely consistent, with several studies failing to detect an association between TL and overall mortality [6–8]. In studies of TL and cause-specific mortality, shorter TL has been linked to higher mortality from cancer, cardiovascular disease, heart disease, and infectious disease [9–13].

Prior studies of the association between TL and mortality have been conducted primarily among populations of European ancestry, with African Americans included in some US studies. However, there is minimal research examining the association between TL and mortality in Bangladeshi or other South Asian populations. Notably, life expectancy is comparatively longer in the US and Europe, where inhabitants live an estimated 79 and 80 years respectively, compared to 72 years in Bangladesh [14, 15].

In this study, we assessed the association between prospectively-measured leukocyte TL and subsequent mortality within three Bangladeshi cohorts: Health Effects of Arsenic Longitudinal Study (HEALS), HEALS Expansion (HEALS-E) and Bangladesh Vitamin E and Selenium Trial (BEST). We used data on >700 deaths occurring among >27,000 participants. These cohorts were originally designed to study arsenic exposure and prevention of arsenic-related disease, but arsenic is not the central focus of this paper. We investigate the association of TL with overall mortality, as well as mortality from chronic diseases, respiratory diseases, circulatory diseases, and neoplasms (growths indicative of cancer).

## RESULTS

### Subject characteristics

Characteristics of cases and controls in each cohort are summarized in Table 1 (which includes all covariates included in the regression analyses). The distribution of TL in each cohort before and after adjustments is shown in Supplementary Figure 1.

**Table 1:** Characteristics of mortality cases and controls selected from the HEALS, HEALS-E, and BEST cohorts

		HEALS		HEALS-E		BEST	
		Controls	Cases	Controls	Cases	Controls	Cases
**Total (n)**		381	367	143	136	237	241
**Sex**	Male	286 (75.0%)	285 (77.7%)	72 (50.3%)	68 (50.0%)	157 (66.2%)	159 (66.0%)
	Female	95 (25.0%)	82 (22.3%)	71 (49.7%)	68 (50.0%)	80 (33.8%)	82 (34.0%)
**Age (years)**	mean (SD)	48.4 (8.9)	48.9 (9.3)	46.3 (10.4)	46.5 (10.2)	48.8 (10.0)	49.5 (9.8)
**Education (years)**	0	167 (43.8%)	190 (51.8%)	78 (54.5%)	72 (53.0%)	118 (49.8%)	131 (54.3%)
	1-5	106 (27.8%)	87 (23.7%)	35 (24.5%)	38 (27.9%)	65 (27.4%)	57 (23.7%)
	6-16	108 (28.3%)	90 (24.5%)	30 (21.0%)	26 (19.1%)	54 (22.8%)	53 (22.0%)
**Smoking**	Never	125 (32.8%)	94 (25.6%)	69 (48.3%)	60 (44.1%)	123 (51.9%)	102 (42.3%)
	Former	66 (17.3%)	80 (21.8%)	21 (14.7%)	22 (16.2%)	40 (16.9%)	44 (18.3%)
	Current	190 (49.9%)	193 (52.6%)	53 (37.1%)	54 (39.7%)	74 (31.2%)	95 (39.4%)
**Own TV**	Yes	228 (59.8%)	244 (66.5%)	51 (35.7%)	52 (38.2%)	103 (43.5%)	151 (62.7%)
**Own land**	Yes	162 (42.5%)	184 (50.1%)	74 (51.7%)	70 (51.5%)	122 (51.5%)	127 (52.7%)
**Urinary arsenic (ug/g) ^a^**	<87.9	88 (23.1%)	68 (18.5%)	34 (23.8%)	38 (27.9%)	69 (29.1%)	45 (18.7%)
	87.9 - 159.9	98 (25.7%)	66 (18.0%)	39 (27.3%)	33 (24.3%)	51 (21.5%)	27 (11.2%)
	160.0 - 298.2	115 (30.2%)	118 (32.2%)	38 (26.6%)	29 (21.3%)	38 (16.0%)	31 (12.9%)
	>298.2	80 (21.0%)	115 (31.3%)	32 (22.4%)	36 (26.5%)	79 (33.3%)	138 (57.3%)
**BMI (kg/m^2^)**	<17.47	91 (23.9%)	135 (36.8%)	43 (30.1%)	53 (39.0%)	57 (24.1%)	93 (38.6%)
	17.47 - 19.09	101 (26.5%)	93 (25.3%)	34 (23.8%)	23 (16.9%)	56 (23.6%)	51 (21.2%)
	19.10 - 21.53	97 (25.5%)	69 (18.8%)	36 (25.2%)	29 (21.3%)	56 (23.6%)	51 (21.2%)
	>21.53	92 (24.1%)	70 (19.1%)	30 (21.0%)	31 (22.8%)	68 (28.7%)	46 (19.1%)
**Telomere length^b^**	TQI, T/S	0.78 (0.16)	0.77 (0.17)	0.65 (0.13)	0.65 (0.14)	0.63 (0.09)	0.61 (0.11)

^a^ Urinary arsenic is adjusted for creatinine concentration

^b^ T/S ratio is shown for HEALS, TQI is shown for HEALS-E and BEST

The pooled sample is 68% male and the mean baseline age is 48.4 years. Mortality cases tend to have higher arsenic exposure based on arsenic measured in urine [16, 17]. The BEST cohort has the highest mean exposure while HEALS-E has the lowest. As expected, in the combined case-control sample TL was inversely associated with age in HEALS (ß=−0.034, P<2×10^−16^), HEALS-E (ß=−0.034, P=1.4×10^−8^), and BEST (ß=−0.030, P=2.6×10^−11^). TL was not significantly associated with sex in HEALS, HEALS-E, or BEST.

### Association between TL and mortality

The associations between TL and mortality outcomes were estimated using conditional logistic regression and are summarized in Table 2. In HEALS and BEST, we observed a significant association between shorter TL and increased overall mortality (OR=0.83, P=0.03 and OR=0.79, P=0.03). The inverse association was present in both HEALS and BEST for both mortality from chronic disease (OR=0.80, P=0.01 and OR=0.78, P=0.03) and mortality from circulatory disease (OR=0.77, P=0.03 and OR=0.73, P=0.04). In HEALS-E, TL was not significantly associated with any of the mortality outcomes.

**Table 2:** Odds ratios (ORs) for the association between telomere length and subsequent mortality in the HEALS, HEALS-E, and BEST cohorts

	Mortality Outcome	Case/Control	OR^a^	95% CI	P-Value
HEALS	overall	367/381	0.83	0.70, 0.98	0.03
	chronic disease	297/381	0.80	0.67, 0.95	0.01
	circulatory disease	143/381	0.77	0.61, 0.97	0.03
	neoplasm	64/381	0.86	0.63, 1.16	0.32
	respiratory disease	49/381	0.70	0.47, 1.05	0.09
HEALS-E	overall	136/143	1.03	0.80, 1.34	0.82
	chronic disease	112/143	1.14	0.86, 1.50	0.36
	circulatory disease	53/143	1.08	0.75, 1.56	0.66
	neoplasm	23/143	1.22	0.71, 2.09	0.48
	respiratory disease	23/143	1.86	0.92, 3.79	0.08
BEST	overall	241/237	0.79	0.64, 0.97	0.03
	chronic disease	207/237	0.78	0.63, 0.97	0.03
	circulatory disease	84/237	0.73	0.54, 0.98	0.04
	neoplasm	77/237	0.79	0.58, 1.07	0.13
	respiratory disease	17/237	1.11	0.46, 2.67	0.81
Pooled	overall	744/761	0.86	0.77, 0.96	0.0082
	chronic disease	616/761	0.85	0.76, 0.96	0.0075
	circulatory disease	280/761	0.81	0.69, 0.95	0.0081
	neoplasm	164/761	0.88	0.72, 1.06	0.16
	respiratory disease	89/761	0.95	0.72, 1.25	0.71

^a^ ORs correspond to a one standard deviation difference in telomere length

**Figure 1:**
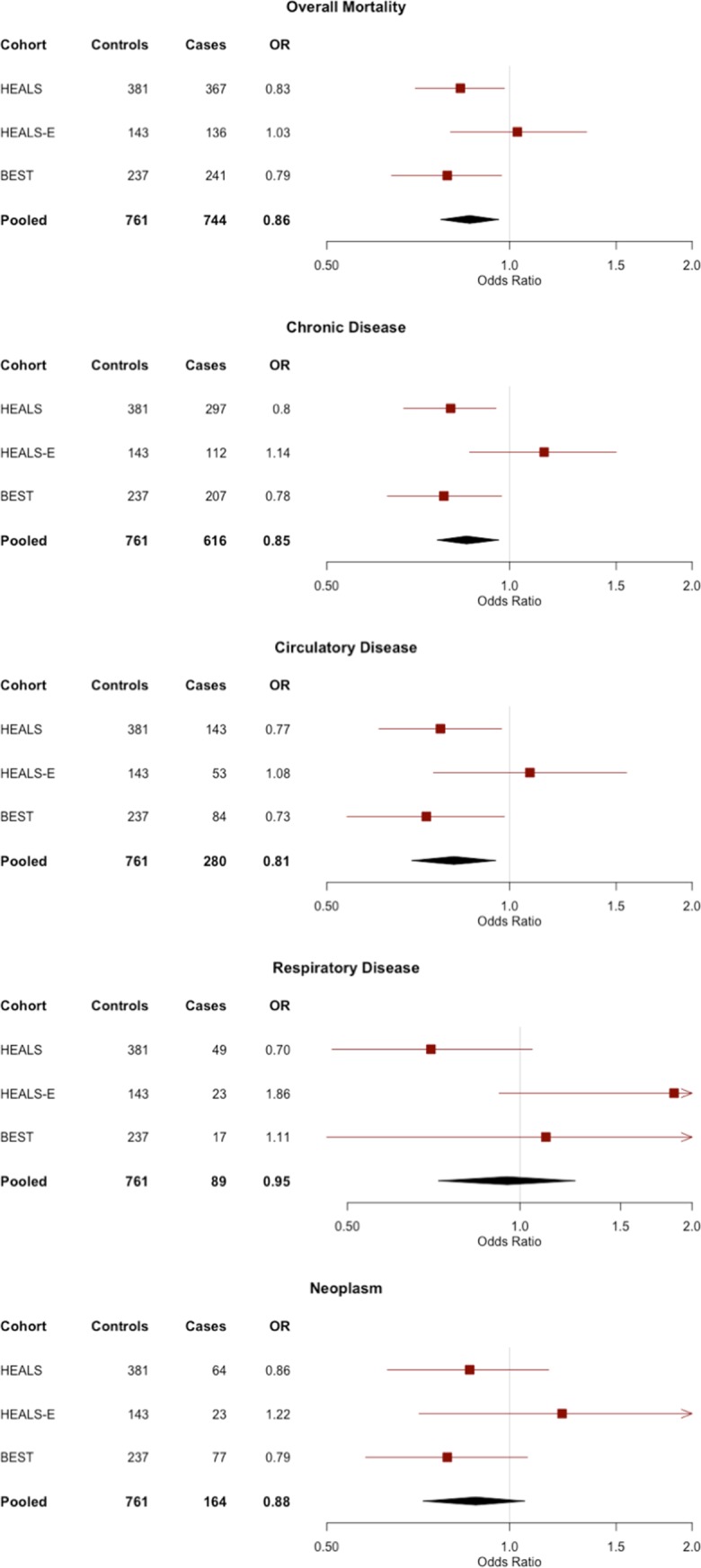
Forest plots of the odds ratios (ORs) and 95% confidence intervals for the association between telomere length and mortality in HEALS, HEALS-E, and BEST cohorts ORs correspond to a 1 standard deviation difference in telomere length.

**Figure 2:**
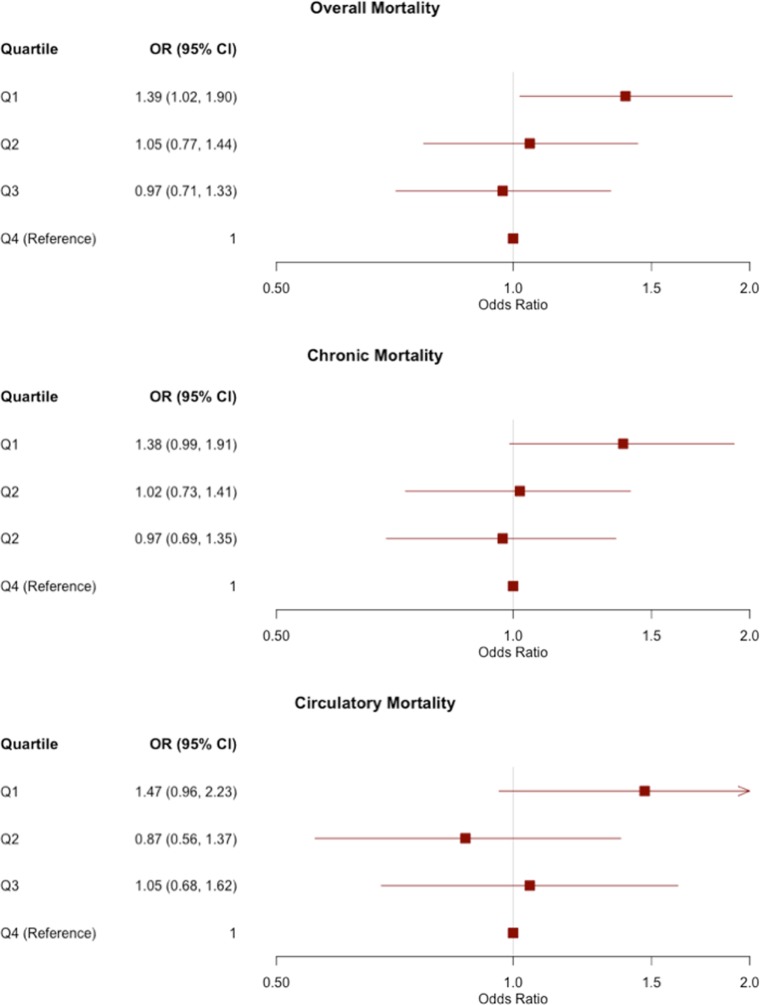
Odds ratios (ORs) and 95% confidence intervals for the association between TL quartiles and mortality outcomes in pooled analyses of all three cohorts TL is divided into quartiles based on the distribution in controls. The reference group is the longest quartile of TL. The OR corresponding to each quartile compares the odds of mortality of the given quartile to the odds of mortality in the longest quartile.

In the pooled analysis, shorter TL was significantly associated with overall mortality (OR=0.86, P=0.008), mortality from chronic disease (OR=0.85, P=0.008), and mortality from circulatory disease (OR=0.81, P=0.008). The point estimates and confidence intervals of each outcome are shown in forest plots across all cohorts in Figure 1. For the three mortality outcomes showing an association with TL (P<0.05) in pooled analyses, the association between TL quartiles and mortality is shown in Figure 2. For each outcome, the shortest TL quartile shows the most prominent association with increased mortality.

TL did not have significant interactions with sex, age, BMI, smoking, or arsenic exposure in any pooled models.

### Assessment of collider bias

Because all BEST participants have arsenical skin lesions at baseline (which is part of the eligibility criteria for BEST), we risked introducing collider bias into our analysis by effectively conditioning on skin lesion status (i.e., conducting our analysis among skin lesion cases only). Skin lesion status may act as a collider because skin lesion risk is affected by arsenic exposure and may also be affected by TL (i.e., our unpublished work suggests that individuals with short TL have higher skin lesion risk, but arsenic does not have a detectable impact on TL). Using very large simulated data sets, we were able to identify the expected direction of the collider bias introduced under the causal model described in Supplementary Figure 2.

Based on analyses of simulated data, the association between TL and mortality would be biased towards higher beta values in the SL subset as compared to a random sample of the population (Supplementary Table 1). We are only able to detect this bias when we simulate data with very large effect sizes and very large sample sizes. In our main analysis, we observe an inverse association between TL and mortality, and because this association is detected even in the presence of a potential bias towards the null, collider bias is not likely to be responsible for the association between TL and mortality observed in BEST.

## DISCUSSION

In this study of leukocyte TL and subsequent mortality in three Bangladeshi cohorts, we observed an association between shorter TL and increased overall mortality in analyses pooled across all three cohorts. In two of the cohorts (HEALS and BEST), we found a significant, inverse association between TL and overall mortality, mortality from chronic disease, and mortality from circulatory disease.

Several aspects of this study are unique. This is the first study of TL and subsequent mortality in a South Asian cohort, with prior studies being conducted almost entirely among individuals of European ancestry [1]. Thus, our data provides compelling evidence that the TL-mortality association observed in prior studies is generalizable to a rural South Asian population with substantially different environmental exposures, lifestyle profiles, and genetic backgrounds as compared to prior studies. In addition, most studies of the association between TL and mortality have been conducted using older populations; the relationship between TL and mortality may be different among populations of different age because TL may be associated with a number of age-related diseases and conditions that vary in incidence across age groups [18]. Studies also suggest that older populations have less variability in TL [1]. An additional strength of our study is confounder adjustment. Most prior studies of TL and mortality report analyses that are minimally adjusted for important covariates, with adjustments for only age, sex, and sometimes race [1]. Our models adjust for a number of additional socioeconomic, lifestyle, and health related covariates, further strengthening the case that TL is causally related to mortality.

In BEST, HEALS, and pooled samples, the observed association between shorter TL and circulatory death is particularly interesting in light of previous research. A recent meta-analysis including 43,725 participants found a consistent association between TL and cardiovascular disease risk in both prospective and retrospective studies [19], and Mendelian randomization studies suggest that this association is causal [13, 20, 21]. In contrast, while prior studies have found associations between short TL and cancer mortality, the association between TL and cancer risk is inconsistent [22] and Mendelian randomization studies have not provided strong evidence that short TL is a causal risk factor for any specific type of cancer [20–22].

While HEALS (and HEALS-E) can be viewed as population-based cohorts [16], BEST includes only subjects who had arsenical skin lesions at the time of enrollment [23]. We performed a simulation to confirm that the selection of skin lesion cases only into the BEST cohort was unlikely to induce the association between TL and mortality observed in BEST as a consequence of collider bias[24].

It is unclear why HEALS-E does not show a significant association between shorter TL and mortality while HEALS and BEST do. One possibility is that analyses of HEALS-E lack the power to detect an association because of small sample sizes. In our analyses of overall mortality, the 95% confidence intervals overlap for all three cohorts, and it is possible that apparent differences across cohorts are due to sampling error. HEALS-E also has lower arsenic exposure than the other two cohorts. It is possible that the effect of TL on mortality is more pronounced in populations with high arsenic exposure, although a TL-arsenic interaction was not observed in pooled analyses. BEST is also slightly older than HEALS and HEALS-E. In the full cohorts, the mean age is approximately 42 in BEST compared to 37 in both HEALS and HEALS-E. However, in the analyzed cases and controls, the mean ages of HEALS, HEALS-E, and BEST are all 50 years, so it appears unlikely that age differences contribute to differences in the TL-mortality association observed across cohorts.

We have measured average telomere content in this study using two different technologies: qPCR (for HEALS) and our Luminex-based method (QGP, for HEALS-E and BEST). While both methods are less precise than Southern Blot, the gold standard for average TL measurement, we have shown that both methods are reproducible and show a clear correlation with Southern Blot. The Southern Blot method is not feasible for a number of reasons, including high DNA input requirements, higher costs, and lower throughput. In order to harmonize these two different types of measures for pooled analyses, we converted both to standard normal variables and adjusted for batch effects.

In this study, we combined data on deaths across three cohorts to obtain a sample size that is larger than many prior studies on this topic. However, future studies of the association between TL and mortality in Bangladesh would benefit from a larger sample size, which would provide more precise association estimates, particularly for analyses of cause-specific mortality, which currently have small sample sizes and, low power for detecting weak associations. As additional deaths occur in these cohorts, we will be able to perform larger follow-up studies of the TL-mortality association.

## MATERIALS AND METHODS

### Study participants

HEALS is a prospective cohort study of 11,746 married men and women from Araihazar, Bangladesh. Participants were recruited between October 2000 and May 2002. Demographic data, lifestyle data, and blood samples were collected at baseline interviews. HEALS was designed to evaluate the long and short-term effects of arsenic consumed in drinking water and has been described extensively elsewhere [16]. The HEALS-E cohort was establishing using the same methods as HEALS, and participants were recruited from the same area from 2006 to 2008, with 8,287 total participants. Informed consent was obtained from all participants. The study procedures were approved by the University of Chicago and Columbia University Institutional Review Boards and the Ethical Committee of the Bangladesh Medical Research Council.

BEST is a randomized chemoprevention trial of 7,000 participants from Araihazar, Matlab, and surrounding areas. All participants have skin lesions associated with arsenic exposure. The study was created to investigate the effects of vitamin E and selenium on non-melanoma skin cancer risk and oxidative stress [23]. Participant randomization was initiated in 2006. Demographic and lifestyle data and blood samples were collected at baseline. The study procedures were approved by the Institutional Review Boards of Columbia University and the University of Chicago and the Ethical Committees of the Bangladesh Medical Research Council and the International Center for Diarrhoeal Disease Research, Bangladesh (ICDDR,B).

### Ascertainment of deaths

In HEALS, mortality was evaluated at biennial in-person visits from 2000-2009. Cause of death was established using a verbal autopsy questionnaire validated by the ICDDR,B. Questionnaires were administered and reviewed by trained study physicians who determined a cause of death. Cause of death was coded using the World Health Organization’s tenth revision of the International Classification of Diseases (ICD-10) [25]. Deaths were ascertained in a similar way in BEST, but with semi-weekly household visits to alert study physicians to participant deaths [25].

Deaths from chronic disease excluded deaths from parasites and infectious disease (A00-B99), pregnancy and childbirth (O00-O99), and external or self-inflicted causes (S00-Y99). The majority of deaths from chronic disease were due to circulatory disease (I00-I99), respiratory disease (J00-J99), or neoplasms (C00-D49).

### Nested case-control study sampling

To assess the association between TL and subsequent mortality, we conducted nested case-control studies within HEALS, HEALS-E, and BEST, frequency matching mortality cases in each cohort to an equal number of randomly-selected surviving controls based on sex and 5-year age intervals. Because age and sex are associated with TL, age and sex matching our case control groups avoids confounding of the association between TL and mortality by these variables [1]. Sampling was conducted in 2012 and only reflects deaths occurring prior to 2010.

In HEALS we observed 773 deaths during the study period. DNA was available for 426 of these cases, and 426 matched controls for TL measurement. After quality control (QC) exclusions (described below), we included data on 367 HEALS mortality cases and 381 HEALS controls. In HEALS-E we observed 149 deaths. DNA was available for 146 of these cases and 148 matched controls for TL measurement. After QC exclusions, we used 136 cases and 143 controls. From BEST we observed 276 deaths. DNA was available for 271 cases and 242 matched controls. Of these, TL data for 241 cases and 237 controls passed our QC filters.

### Laboratory methods

In the HEALS cohort, DNA was extracted from clotted blood using Flexigene DNA kit (Cat # 51204) from Qiagen, Valencia, USA. In the HEALS-E and BEST cohorts, DNA was extracted from whole blood using QIAamp 96 DNA Blood Kit (cat# 51161) from Qiagen. Samples’ quality was checked using a Nanodrop 1000.

For HEALS, quantitative polymerase chain reaction (qPCR) was used to measure the abundance of the telomere repeat sequence in relation to a two-copy gene (RPLP0). Lab technicians were blinded to demographic and most health information of the subjects, and case and control samples were randomized across plates and well positions. We used a qPCR protocol as described by Ehrlenbach et al [7]. Briefly, ninety-six well plates were loaded with six replicates of the subject sample: three for measuring telomere content and three for measuring the reference gene (RPLP0). A reference sample was included on each plate (six replicates per plate) to account for inter-plate variation.

The PCR was performed using 30 μL amplification reaction volume of 1× Qiagen Quantitect Sybr Green Master Mix (Qiagen) and 25 ng of template DNA. For the telomere sequences, the primer sequences used were 5′GGTTTTTGAGGGTGAGGGTGAGGGTGAGGGTGAGGGT3′ (forward) and 5′TCCCGACTATCCCTATCCCTATCCCTATCCCTATCCCTA3′ (reverse). For the reference sequence, the primer sequences used were 5′CAGCAAGTGGGAAGGTGTAATCC3′ (forward) and 5′CCCATTCTATCATCAACGGGTACAA3′ (reverse). Plates were run at 95°C for 15 minutes to denature the DNA. This was followed by 25 cycles of 95°C for 15 seconds, 54°C for 2 minutes, and 72°C for 1 minute. Plates were then left at 72°C for 4 minutes.

We extracted a C_t_ (cycle threshold) value from the qPCR data for each sample. We used the C_t_ to calculate a T/S ratio comparing the abundance of telomere sequence and the reference gene sequences on each plate. By including references on each plate, we standardized this measure across all samples. A sample’s T/S ratio is proportional to its relative TL [7]. T/S ratio was calculated using the following equation:

$$\frac{T}{S}\text{ratio}=\frac{2^{{C_{t}}_{{\text{telomere}}^{\left(\text{reference}\right)}}}/2^{{C_{t}}_{{\text{telomere}}^{\left(\text{sample}\right)}}}}{2^{{C_{t}}_{{\text{RPLP}0}^{\left(\text{reference}\right)}}}/2^{{C_{t}}_{{\text{RPLP}0}^{\left(\text{sample}\right)}}}}$$

where C_t_ is the cycle threshold.

For QC purposes, a coefficient of variation (CV) was calculated for each set of telomere and RPLP0 replicates using the following equation:

$$\text{CV}=100\times \frac{\sqrt{\frac{{\sum {(x}_{i}-\overset{-}{x})}^{2}}{n}}}{\overset{-}{x}}$$

For telomere and RPLP0 triplicates measured in subject samples, we excluded a single measurement if it caused the CV to rise above 3 percent or 1 percent respectively. If after this exclusion the CV was still above the thresholds, the subject was excluded from our analysis. For telomere and RPLP0 sextuplicates measured in reference samples, we excluded up to two measurements if they caused the CV to rise above the same 3 percent or 1 percent thresholds. If after this exclusion the CV was still above the thresholds, the whole plate was excluded from our analysis. Outliers, defined as measurements more than three standard deviations away from the mean of either the raw measurement (C_t_) or T/S ratio, were excluded.

We verified reproducibility of our telomere measurements by re-running 37 samples on separate days. The overall pair-wise CV between the original and later measurements of these samples was 11.7 percent. The pairwise CVs were calculated using the following equation:

CV=100×∑(x1−x2)22n∑(x1−x2)2n

In the HEALS-E and BEST cohorts, TL was measured using Luminex QuantiGene Plex (QGP) assay [26]. This method is strongly correlated with the Southern blot TL measures and is an accurate and reproducible method for measuring TL [26, 27].

The QGP assay measures telomere sequence abundance in relation to a two-copy reference gene (ALK). The QGP protocol and its accuracy and precision have been described previously [26–28]. In summary, fluorescent Luminex microbeads were equipped with capture probes, which cooperate with several additional probes (capture extenders, label extenders, and blockers) to bind the telomere repeat sequence or the reference gene sequence. A pre-amplifier probe then binds to the label extender, and allows for the binding of biotinylated amplifiers and fluorescent labels, whose abundance can be measured by a Luminex instrument. The fluorescence intensity is proportional to the number of target sequences. The Telomere Quantity Index (TQI) for each sample is the ratio of telomere DNA measured to reference DNA measured and accounts for inter-plate variation by running a reference sample on every individual plate [26].

In a prior study, Pierce et al. calculated the CVs for replicate QGP TL measures which ranged from 5.4 to 9.1 depending on the method of calculation [27]. We assume our measurements, which followed the same laboratory protocol, are similarly reproducible. Pierce et al. found that 14% of the variation in TL was attributable to age. Our study found a comparable 10% of variation attributable to age.

Our qPCR and QGP methods of telomere measurement both produced a ratio of telomere abundance to reference gene abundance. This ratio is expressed relative to a reference DNA sample. We standardized the TL measures within each cohort to a mean of 0 and a variance of 1.

### Adjustments to TL measures

Because qPCR measures tend to vary according to plate and well position, we further attempted to remove this variation from our TL measurements using mixed linear models. We created a mixed effects model predicting TL with plate as the random effect (106 plates) and well position as the fixed effect (14 well positions). The residuals produced by this model serve as an estimate of TL with experimental variation removed. We standardized the residuals to a mean of 0 and standard deviation of 1 and then included them in the models as our TL measurement.

We did not adjust for well position in HEALS-E and BEST samples because, unlike qPCR, QGP does not show well position effects [28]. We removed any effect of plate on QGP TL using a linear regression; we predicted TL with categorical variables representing “plate” as predictors (52 plates) and then removed plate’s effect on TL.

### Statistical methods

All analyses accounted for the following covariates: Age (years), sex (male or female), body mass index (BMI) quartiles (<17.5, 17.5-19.1, 19.1-21.5, >21.5), smoking status (never, former, current), arsenic concentration in urine quartiles (creatinine-adjusted urinary arsenic) (<87.87, 87.87-159.9, 160.0-298.2, >298.2), years of formal education (0, 1-5, 6-16), land ownership, and television ownership. BMI and arsenic exposure quartiles were based on the distribution among controls pooled across all cohorts.

We estimated the association between TL and mortality using conditional logistic regressions in order to account for the matched case-control design. Regressions were conditioned on the sex-specific, five-year age strata used to match controls to cases. In instances where within-strata sample sizes were too small to allow for matching, we combined adjacent age strata within sex categories.

Each cohort was analyzed separately for overall mortality and for mortality due to chronic disease, respiratory disease, circulatory disease, and neoplasm. In each cohort, subjects with TL measurement were discarded if missing data on covariates included in the model. We also conducted pooled analyses including all cases and controls from HEALS, HEALS-E, and BEST, adjusting for cohort as a categorical variable. In these pooled analyses we conducted interaction tests for TL with the following mortality-related covariates: age, sex, BMI, smoking, and arsenic exposure.

### Simulation to assess potential collider bias

All BEST participants have arsenical skin lesions. Because skin lesion risk is effected by arsenic exposure and may be effected by TL, skin lesion status may act as a collider. We ran a simulation to measure any potential bias introduced by conditioning on skin lesion status in BEST. By simulating a very large dataset, we were able to determine the expected direction of the potential collider bias under the causal model described in Supplementary Figure 2. We simulated 100 datasets of 1,000,000 observations and four variables: TL (W) and binary variables representing arsenic exposure (X), skin lesion status (Y) and mortality (Z). From each simulated dataset we additionally sampled 250 mortality cases and 250 mortality controls so that we could simultaneously simulate what we would expect to see in a dataset the size of our BEST data. We did this for both the overall dataset and the subset of SL cases. TL was generated as a random standard normal variable. Arsenic was generated as a random binary variable with a 0.3 probability of being high exposure (x=1). The skin lesion (Y) and mortality (Z) risk were influenced by W and X. Y and Z were generated as random binary variables using the following equations to determine their respective probabilities of a case:

$$P(y=1)=\frac{1}{1+e^{-1*({ß}_{y0}+\left({ß}_{yw}*W\right)+\left({ß}_{yx}*X\right))}}$$

$$P\left(z=1\right)=\frac{1}{1+e^{-1*({ß}_{z0}+\left({ß}_{zx}*X\right))}}$$

We repeated the simulation three times with weak effect sizes similar to what we observed in our data, moderate effect sizes, and strong effect sizes. The effects of arsenic exposure on skin lesion and mortality (${ß}_{YX}$ and ${ß}_{ZX}$) were set to 0.405, 0.833, and 1.609 (OR=1.5, 2.3, and 5), respectively. ${ß}_{YW}$ was set to -0.289, -0.693, and -0.1609 (OR=0.75, 0.5, and 0.2), and ${ß}_{ZW}$ was set to 0 (OR=1) in all simulations.

We defined the baseline risk of skin lesions to be 5 percent (${ß}_{Y0}=-\text{log}((1-0.05)/0.05))$ and baseline risk of mortality to be 3 percent (${ß}_{Z0}=-\text{log}((1-0.03)/0.03))$. The association between TL and mortality was estimated using a simple logistic regression for the entire sample and just skin lesion cases.

## SUPPLEMENTARY MATERIAL


