# Supplementary material for: The association between telomere length and mortality in Bangladesh
Source: Aging (Albany NY). 2017 Jun 15;9(6):1537–48. doi: 10.18632/aging.101246 (PMC5509454; doi:10.18632/aging.101246)
Supplement: Supplementary file 1 [file aging-09-1537-s001.pdf]

SUPPLEMENTARY MATERIAL

Supplementary Table 1. Association between telomere length and mortality in simulated datasets generated according to the causal model in Supplementary Figure 2.

Weak Effects:

Effect size of arsenic and TL on SL: $OR_{ARSENIC}$ = 1.5, OR_{TL} = .75

Effect size of arsenic and TL on Mortality: $OR_{ARSENIC}$ = 1.5, OR_{TL} = 1

		Median Beta	Median P-Value
Population of 1,000,000	Full dataset	$5.7x10^{-7}$	0.59
	Restricted to skin lesion cases	$-1.9x10^{-5}$	0.43
250 cases, 250 controls	Sampled from full dataset	$-2.3x10^{-3}$	0.48
	Sampled from skin lesion cases	$2.3x10^{-4}$	0.53

Moderate Effects:

Effect size of arsenic and TL on SL: $OR_{ARSENIC}$ = 2.3, OR_{TL} = .5

Effect size of arsenic and TL on Mortality: $OR_{ARSENIC}$ = 2.3, OR_{TL} = 1

		Median Beta	Median P-Value
Population of 1,000,000	Full dataset	$-5.4x10^{-6}$	0.50
	Restricted to skin lesion cases	$5.2x10^{-4}$	0.43
250 cases, 250 controls	Sampled from full dataset	$-4.8x10^{-3}$	0.48
	Sampled from skin lesion cases	$5.2x10^{-3}$	0.46

Strong Effects:

Effect size of arsenic and TL on SL: $OR_{ARSENIC}$ = 5, OR_{TL} = .2

Effect size of arsenic and TL on Mortality: $OR_{ARSENIC}$ = 5, OR_{TL} = 1

		Median Beta	Median P-Value
Population of 1,000,000	Full dataset	$-3.3x10^{-6}$	0.45
	Restricted to skin lesion cases	$1.1 x10^{-2}$	$9.1x10^{-4}$
250 cases, 250 controls	Sampled from full dataset	$-3.5x10^{-3}$	0.47
	Sampled from skin lesion cases	$3.7x10^{-2}$	0.16

Data was simulated with no effect of TL on mortality (i.e., beta equals zero). Analyses of simulated data restricted to skin lesion cases are meant to demonstrate the potential impact of collider bias on the beta estimate obtained from BEST. For each scenario, we simulated 100 independent replicate datasets.

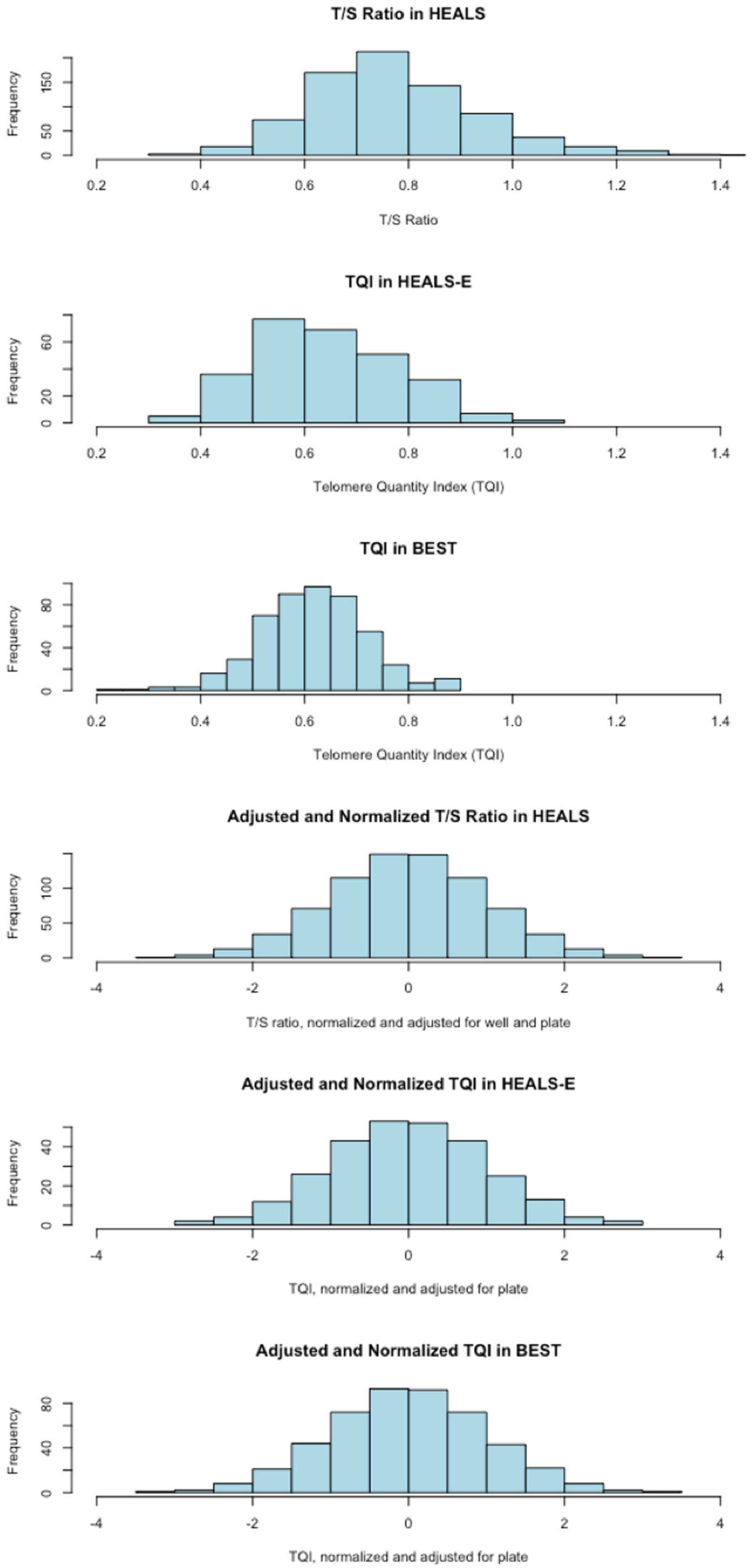

Supplementary Figure 1. Distribution of TL before (first three histograms) and after adjustments (last three histograms). Adjustments are described in detail in the Methods section.

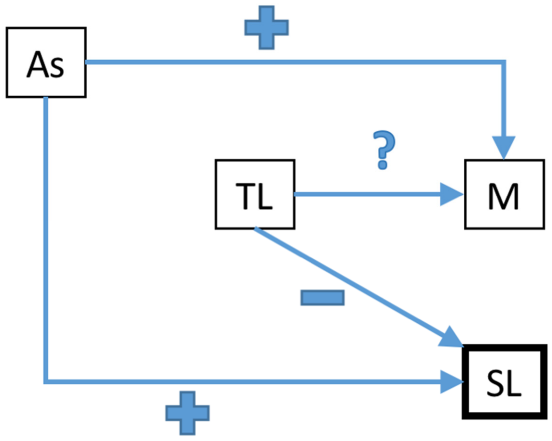

Supplementary Figure 2. Causal model used to simulate the datasets used to demonstrate the potential effect of collider bias on the association between TL and mortality -- including variables arsenic (As), telomere length (TL), mortality (M), and skin lesions (SL). Positive associations are represented by (+) while negative associations are represented by (-).
